# Ultrafast Optical Hydrogen Modulator in a Palladium Hydride Nanoparticulate Thin Film

**DOI:** 10.1002/nap2.70288

**Published:** 2026-09-06

**Authors:** Floriana Morabito, Paula H. Aguado, Luca Pedicone, Alessandro Baserga, Andrés R. Botello Méndez, Stefano Dal Conte, Christoph Gadermaier, Giulio Cerullo, Zeila Zanolli, Marcel Di Vece

**Affiliations:** ^1^ Dipartimento di Fisica Politecnico di Milano Milano Italy; ^2^ Debye Institute for Nanomaterials Science Utrecht University Utrecht the Netherlands; ^3^ Interdisciplinary Centre for Nanostructured Materials and Interfaces (CIMaINa) and Physics Department “Aldo Pontremoli” Università degli Studi di Milano Milan Italy

**Keywords:** DFT, hydride, optical switching, palladium, thin film, ultrafast spectroscopy

## Abstract

Electro‐ and thermochromic materials change their optical properties through the physical movement of atomic species within thin films. This movement is induced by electric fields or high temperatures and occurs on the second to minute timescale. Ultrashort laser pulses can manipulate atomic positions on the femto‐ to picosecond timescale. In this work, we modulated the optical properties of a palladium hydride nanoparticulate thin film on the picosecond timescale using femtosecond optical pulses. Density functional theory calculations confirmed that the incident photons activate interstitial hydrogen atoms and the subsequent trajectories of these atoms affect the dielectric properties of the palladium hydride thin film. These responsive interstitial hydrogen atoms provide a modulator for optical switching on the picosecond timescale and could lead to faster photochromic materials.

## Graphical abstract

An all‐optical palladium metal‐hydride modulator was investigated with ultrashort laser pulses, which can manipulate hydrogen positions on the femto‐to picosecond timescale. The incident photons activate interstitial hydrogen atoms and their subsequent trajectories affect the dielectric properties of the palladium hydride thin film, providing optical switching on the picosecond timescale that could lead to faster photochromic materials.

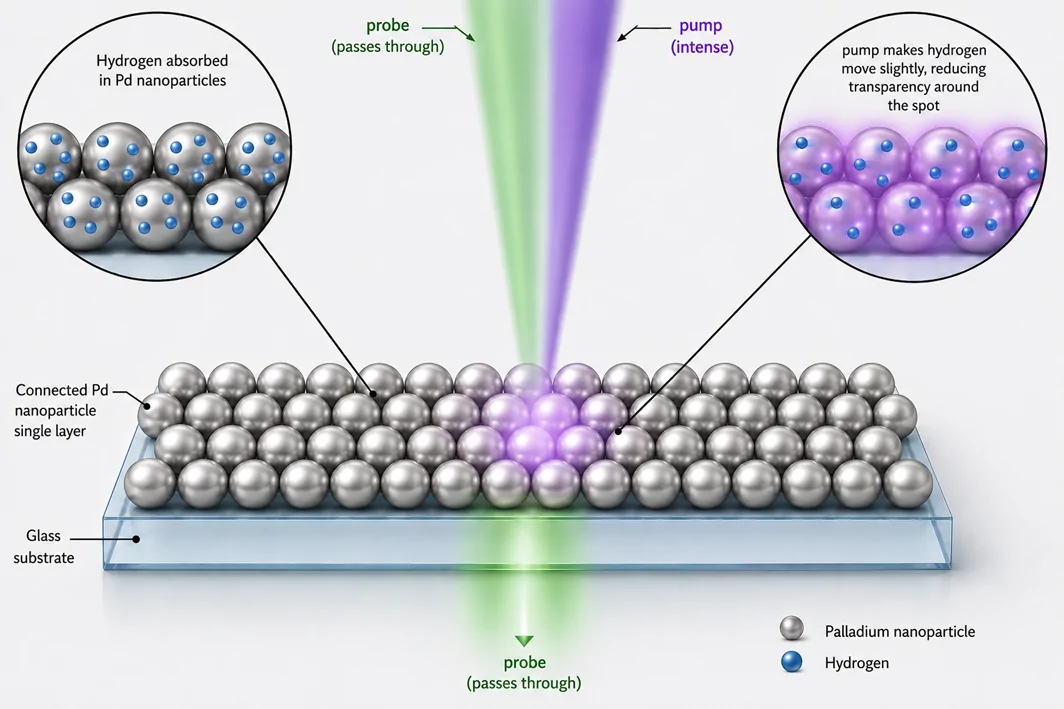

## 1 Introduction

Since the discovery that palladium can absorb large quantities of hydrogen [1], metal hydrides have been a continuing topic of study. Hydrogen storage in metals is an attractive research topic aimed at clean energy and has led to the development of the metal hydride battery [2, 3]. Subsequent studies of the metal‐hydrogen system showed that hydrogenation has a strong influence on the electronic density of states of various metals [4] because hydrogen atoms tend to attract the weakly bound electrons of the metal [4], inducing a metal‐insulator transition [5]. One of the most striking visual transformations of a metal hydride is the “switchable mirror”, which is a metal‐insulator transition in a thin yttrium film upon hydrogen absorption [6, 7]. These hydrogen‐induced optical transformations are of significant interest to nanophotonics with metal hydrides [8, 9]. In photochromic yttrium trihydride, this metal‐insulator transition can be reversed by an incident laser pulse, reducing transmittance over time scales of hours [10].

Due to its noble character, which makes it easy to handle, palladium (Pd) and its hydride (PdH) have been extensively studied in the literature [11, 12]. After hydrogen intercalation, two new peaks appear in the local density of states (LDOS) below the Fermi energy: one below the d‐band, and the other one well below the Fermi energy level due to the hybridisation between the Pd 4d and the H 1s states. The hybridised bonding states (which are significantly weaker than localised covalent bonds) are lowered in energy because the electrons feel the attractive potential of the additional proton introduced by the hydrogen, with a strong *s* character. The antibonding states created by hybridisation produce the LDOS peak above the Fermi level. The electron carried by the H atom fills the d‐bands near the Fermi level. The total number of states of palladium and hydrogen below the highest‐energy subpeak in the d‐band remains unchanged, but the Fermi level shifts above the d‐bands in PdH [12].

These changes in the electron DOS upon hydrogenation modify the dielectric properties of palladium, which have been extensively explored with respect to optical and plasmonic hydrogen detection [9, 13, 14, 15, 16, 17, 18]. The change in the bulk plasmon resonance induced by hydrogenation was used to investigate the structural phase transitions in palladium nanoparticles [19].

Ultrafast optical spectroscopy is primarily used to investigate fast electron excitations and their subsequent phonon generation and charge transfer mechanisms [20, 21, 22, 23]. However, impulsively driven coherent vibrational processes in the electronic ground state [24, 25, 26] have been shown to stimulate atomic motion. Such ultrafast optical manipulation of atomic positions has the potential to harness hydrogenation‐induced photonic switching on the femto‐ to picosecond timescale.

In this work, we propose a new paradigm for ultrafast all‐optical switching based on interstitial hydrogen motion and its influence on the optical spectrum of hydrogenated palladium. We use ultrafast optical spectroscopy to study the photoinduced optical changes in hydrogenated palladium. We investigate a palladium layer that was partially filled with hydrogen atoms. We find that an ultrashort pump pulse triggers the movement of interstitial hydrogen atoms to vacant positions [27], generating changes in the optical absorption spectrum that are detected by a time‐delayed probe pulse. Our experimental results are supported by Density Functional Theory (DFT) calculations that study the effect of the moving interstitial hydrogen on the optical properties.

## 2 Results and Discussion

Palladium nanoparticulate thin films with a nominal thickness of 20 nm were thermally evaporated at the melting temperature of ∼1400°C (∼1 min) onto fused silica substrates, and hydrogenation was performed using ∼1 bar of helium gas containing 15% hydrogen. Figure 1a reports the morphological characterisation of the evaporated palladium nanoparticulate thin films by scanning tunnelling microscopy (STM) and displays the typical nanogranular (nanoparticulate) growth mode previously reported [28], which provides crystallinity and does not affect the hydrogen metal lattice dynamics [9]. The slight increase in size of the palladium nanoparticle grains after hydrogenation (Figure 1b), is likely caused by Ostwald ripening induced by hydrogen [29]. Although it is known that epitaxial palladium thin films can become clamped to the substrate, affecting their thermodynamics, such as formation enthalpy and solubility [30, 31], with concomitant optical changes [32], the nanostructure of the palladium nanoparticulate thin film in this work allows relaxation of the stress originating from clamping and largely mitigates such effects. The experimental optical absorbance spectra, reported in Figure 1c and dominated by a broad plasmon resonance peaked at 300 nm for pristine palladium, show a clear decrease in absorbance upon hydrogenation, as confirmed by the simulations. This bleaching of the static optical absorption spectrum of palladium nanoparticulate thin films upon hydrogenation is consistent with previous work [28, 33]. The lower absorbance at longer wavelengths in this work is likely due to a different deposition method (thermal evaporation versus electron beam deposition), which results in smaller nanoparticles and shifts the plasmon resonance to shorter wavelength [8, 34]. Furthermore, the reduced absorbance upon hydrogenation observed in this work, which corresponds to an optical transmission change of several percent, confirms a hydrogen concentration of at least 30 ± 5% [28], with the hydrogen atoms in the octahedral interstitial positions [35, 36]. The hydrogen concentration did not change over the cause of the experiments, as observed from the optical transmission, providing a constant α‐β phase coexistence crystal. In previous work, the formation of nanoparticle palladium hydrides resulted in a core‐shell structure with each a different phase [19, 28]. The palladium hydride phases are well documented [37] and are not expected to modify the interpretation of the optical results. The finite difference time domain (FDTD) simulations reported in Figure 1c show a similar absorbance change upon hydrogenation. The additional absorbance peak at longer wavelengths in the calculated spectra could arise from the surface plasmon modes [38, 39], which are not present in the experimental data due to a slightly different topology or disorder. In the simulations, in fact, a periodic nanoparticle lattice was used. Due to the imperfect periodicity of the samples, the measured absorption spectra depend more strongly on the dielectric function than the simulated spectra, resulting in a bigger difference between the pristine and the hydrogenated sample.

**FIGURE 1:**
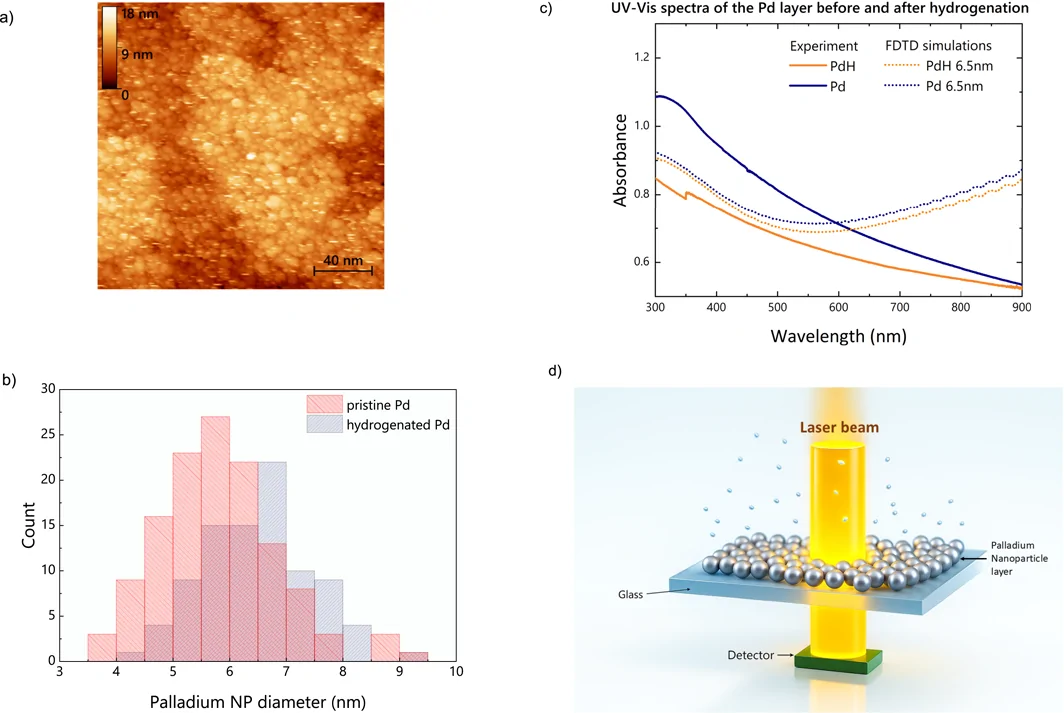
(a) STM image of the pristine palladium nanoparticulate thin film with spherical nanostructures clearly visible as they constitute the Pd film. (b) Palladium grain size distributions in which the hydrogenated Pd nanoparticles (NPs) are centred on a slightly higher value (6.6 ± 0.8 nm) than the one in the pristine state (5.7 ± 0.7 nm). (c) Experimental (solid lines) and simulated (dotted lines) optical transmission spectra for a palladium nanoparticulate thin film consisting of 6.5 nm (aligned with experiments) diameter spherical palladium nanoparticles. The slight difference at the smaller wavelengths indicates plasmonic effects. (d) Schematic depiction of the experimental setup, with grey spheres representing the palladium nanoparticles that constitute the nanoparticulate thin film on a glass support. The disproportionality is for the purpose of clarity.

The ultrafast dynamics of the differential transmission (ΔT/T) of the sample were studied after excitation with an ultrashort visible pump pulse, as shown in the maps of Figure 2a,b. Two time domains can be distinguished: (1) the first few picoseconds, during which the non‐equilibrium electron distribution thermalises via electron‐electron and electron‐phonon scattering and (2) the longer timescale, from ∼4 till 80 ps [40, 41, 42], which is dominated by further relaxation via anharmonic phonon decay and heat transport out of the probed sample volume. The ΔT/T exhibits a broad initial negative signal, that we attribute to reduced metal transmission due to additional conduction electrons excited by the pump pulse. The ΔT/T dynamics, shown in Figure 2c for an exemplary probe wavelength, is superimposed with a strong contribution from coherent lattice oscillations (phonons) [43, 44]. We distinguish two main phonon modes, one with a period of 1–2 ps, which de‐phases within less than two oscillation periods, and one with a period of 26 ps that extends beyond the temporal window of our experiment. The oscillation with the shorter period is attributed to impulsively excited coherent acoustic phonons (most likely the radial breathing mode) in the palladium nanoparticulate thin film, leading to an oscillation in the surface plasmon resonance frequency of the nanoparticle, which produces oscillations in the transient signals. These oscillations have been extensively studied in gold spheres and rods [45, 46, 47]. At zero pump‐probe delay, these oscillations are at a minimum, pointing towards a displacive excitation mechanism, where the electronic excitation shifts the atomic equilibrium positions and the lattice starts oscillating about these new equilibrium positions [48]. These optical phonons cause acoustic phonon de‐phasing and mixing, resulting in reduced amplitude.

**FIGURE 2:**
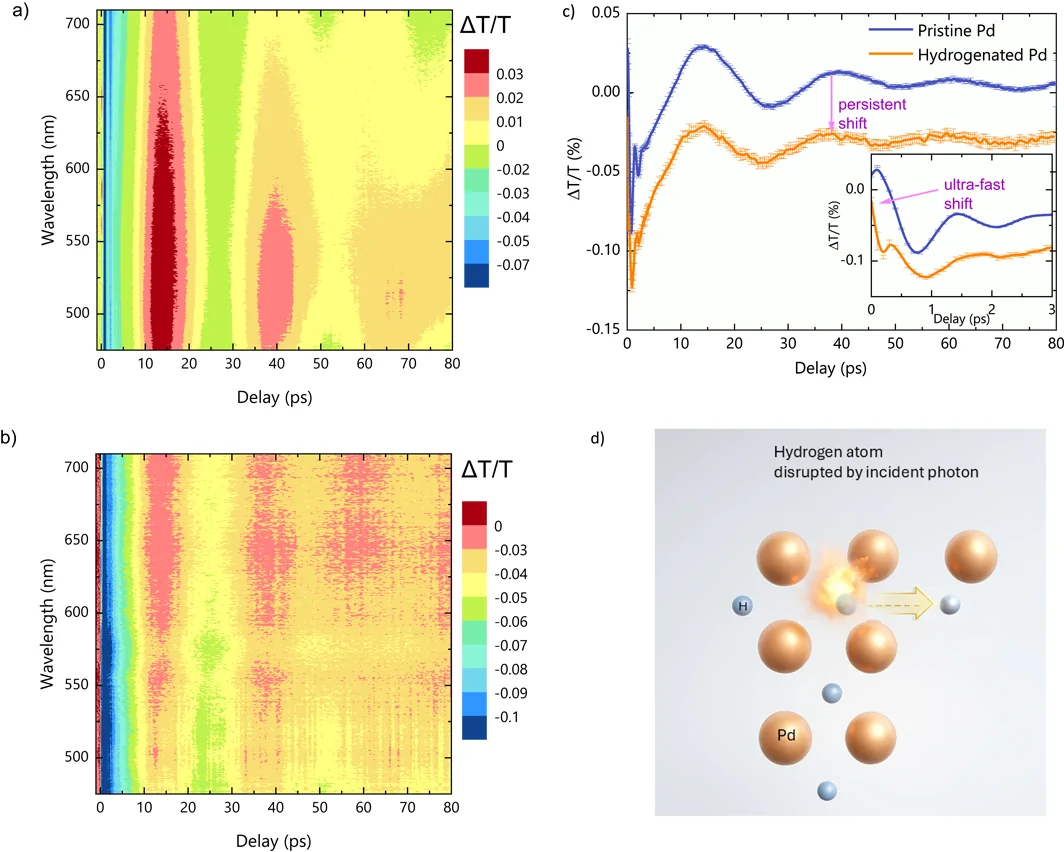
Measured ΔT/T map under 120 μJ cm^−2^ pump laser fluence up to 80 ps after a 510–610 OPA broadband pump pulse for (a) a pristine Pd nanoparticulate thin film, and (b) a hydrogenated Pd nanoparticulate thin film with slightly lower signal around 550 nm due to pump laser light scattering. (c) The ΔT/T dynamics before and after hydrogenation at 650 ± 5 nm clearly illustrates the effect of hydrogen: damping of the oscillations and a negative offset due to the hydrogen‐palladium bond breaking. In the inset the distinct jump of the PdH ΔT/T signal to lower values in the few hundreds of femtoseconds time scale, establishes the electron‐electron interaction origin. (d) Schematic depiction of the effect of an incident photon on the hydrogen‐palladium bond, temporarily broken due to the deposited energy from the photon. This process leads to a short moment of palladium d‐band filling, which translates to a small absorption increase on the nanosecond timescale.

A comparison of two palladium nanoparticulate thin films of slightly different thickness (Supporting Information S1: Figure S1) showed that the strong negative response up to the first 2 ps remained the same, while the period of the long‐lived oscillation is 12 ps in one case and 26 ps in the other [43]. This strong thickness dependence supports their assignment to an acoustic phonon standing wave. Assuming a speed of sound of 3.07 10^3^ m/s, these correspond to wavelengths of 37 and 80 nm, respectively, and suggest a phonon standing wave perpendicular to the film.

While the photoinduced optical response in the pristine sample is spectrally featureless over the probed wavelength range, three main effects are observed upon hydrogenation: (1) the long‐lived oscillations are strongest around 530 nm for the pristine sample and 650 nm for the hydrogenated Pd, (2) the oscillation period decreases from 26 for the pristine sample to 24 ps for hydrogenated Pd and (3) for pristine Pd the non‐oscillatory signal component goes to zero within a few ps, while for the hydrogenated sample a negative ΔT/T ≈ 0.025 signal persists over the entire observation window.

While the dynamics in Figure 2c for the pristine sample have damped oscillations around zero, the dynamics for the hydrogenated sample have damped oscillations of ΔT/T around −0.03%, which lasts beyond the 80‐ps observation window. This negative offset in the palladium hydride nanoparticulate thin film occurs in the initial stages after pumping and indicates a significant, sustained decrease in the optical transmission. In particular, within the first few hundred femtoseconds, hydrogenated palladium exhibits a clear and intense negative ΔT/T (Figure 2c), which, due to its timescale, can only be attributed to electronic excitation. Lower optical transmission could result from electron supply to the conduction band, as observed in yttrium trihydride [10], which strengthens the surface plasmon resonance. Because the low‐lying states in the hydrogen‐palladium alloy are strongly hybridised, a disruption of the hydrogen by the ultrafast pump pulse could lead to movement of the hydrogen to other lattice positions, as schematically depicted in Figure 2d. During this process, the bond between hydrogen and palladium weakens, making palladium d‐electrons available to the surface plasmon resonance on the ps time scale. In an analogous system, hydrocarbon bonds were cleaved heterolytically with the proton 1.24 Å away from its equilibrium position [49]. The “rattling” movement of hydrogen in a palladium hydride brings the hydrogen atoms back to their equilibrium positions at room temperature. This movement occurs on the nanosecond timescales [50, 51], giving ample time for a stable effect, to reach a stable configuration on the ns time scale, and therefore measurable, in the picosecond range with a stable configuration. These time scales are in agreement with the hydrogen atomic jump time τ ∼ 10^−11^ to 10^−9^ s [36, 52], which could be slightly adjusted for the nanoparticulate properties. The laser pulse repetition rate of 2 kHz guarantees a thorough relaxation of this stimulated hydrogen atom movement. A study of photo‐stimulated hydrogen desorption in magnesium nanoparticles shows that light can indeed stimulate hydrogen movement [53]. Furthermore, a light‐induced metal‐insulator transition, measured by electrical conductivity in a yttrium hydride switchable mirror, confirms the electron‐electron interactions after photo‐stimulation [54]. Plasmon resonance activated hydrogen mobility from palladium nanoparticles can even be used for catalytic reactions [55]. The damping of the oscillations for *t* > 4 ps in the palladium hydride is likely also related to the reduced surface plasmon oscillator strength, leading to a weaker interaction between the phonons and the surface plasmon resonance.

At room temperature, the palladium atomic lattice expands by 0.1% for the α‐phase (up to H/Pd ≈ 0.015) and by 3.8% for the β‐phase (H/Pd ≈ 0.7, 1 bar H_2_) with α‐ and β‐phases coexisting in between [56], which is much smaller than for yttrium trihydride, which is often quoted as the reference material for optical changes upon hydrogen absorption [57, 58]. It cannot be excluded that this small volume expansion affects the dynamics of the ΔT/T oscillations directly. The slower relaxation and thermalisation observed in palladium hydride after photoexcitation, compared to pristine palladium, could be attributed to small lattice modifications that influence phonon‐phonon interactions. This could explain why the hydrogenated palladium sample had a non‐zero signal even after 80 ps.

We investigated the ultrafast response of a pristine palladium nanoparticulate thin film for different values of pump fluence and pump and probe wavelengths (Supporting Information S1: Figure S2a–d), revealing a fluence and energy independent response in the visible region. This suggests that the response of the hydrogen atoms saturates already below 120 μJcm^−2^.

To align the simulations, performed with the SIESTA code [59], with the experimental results, a hydrogen concentration of 30% was used. This concentration is in agreement with the static optical absorption measurements shown in Figure 1c and is close to the hydrogen concentration at which significant DOS changes start to occur, as previously reported by Faulkner [60]. Three hydrogen concentrations were calculated: Pd, PdH_0.3_ and PdH with a bulk PdH FCC structure. This is consistent with the coexistence of α‐ and β‐phases with a moderate lattice expansion for a concentration of about 30%, due to the β‐phase [56]. In bulk, hydrogen‐rich β domains nucleate within the α matrix, which also occurs in palladium nanoparticles [19], with phase fractions estimated with the lever rule: *α* ≈ 52%, *β* ≈ 48%. However, coexistence range is considerably reduced at the nanoscale [19, 61], resulting in a large solid‐state solution portion. Because the movement of hydrogen in the β‐phase is likely limited due to the higher energy required for the full occupancy, it is more plausible that interstitial hydrogen movement occurs at phase domain boundaries. Assuming that the primitive cell of PdH contains two hydrogen atoms and two palladium atoms, a supercell was created for the simulations to achieve global 30% occupancy, appreciating the phase domain boundary, with periodically repeated hydrogen vacancies (Figure 3a–c). First, the supercell used four hydrogen atoms, leading to a PdH_0.25_ structure. Then, a fifth H atom was included to model PdH_0.3_. The lowest‐energy atomic configuration for PdH_0.25_, a 2 × 2 × 2 super cell containing 16 Pd atoms, was generated considering all the possible combinations of the four hydrogen atoms (1820 resulting structures [62]).

**FIGURE 3:**
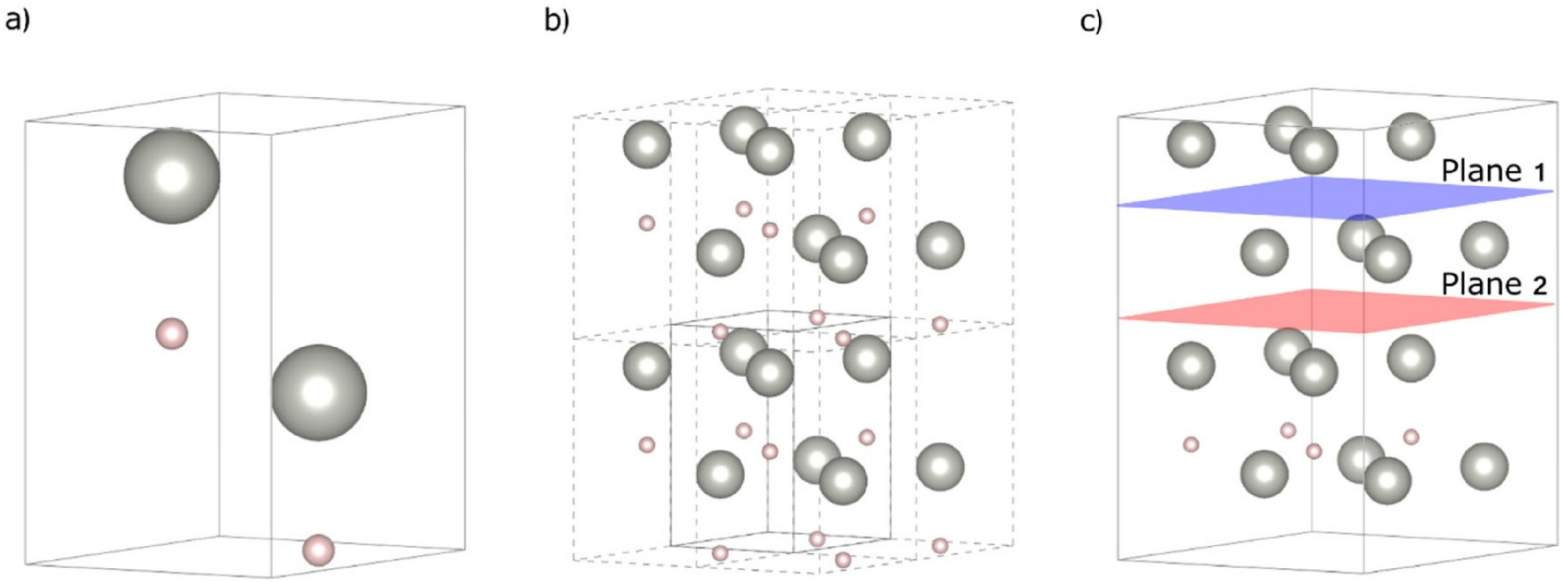
(a) Relaxed calculated structure of PdH_0.25_, (b) PdH Primitive cell and (c) PdH_0.25_ supercell (2 × 2 × 2). Palladium atoms (silver) and Hydrogen atoms (pink).

The calculated band structures of pristine palladium and palladium hydride (25% and 100%), reported in Figure 4a–c, show that the studied systems have metallic behaviour as some of the bands cross the Fermi level. Therefore, the electronic transitions between occupied and empty bands are expected to be continuous, leading to continuous absorption spectra. Despite the increased number of electrons, the calculated Fermi energies consistently increase with respect to the energy of the d‐bands (Figure 4a–c) with increasing hydrogen concentration, in contrast with previous calculations [63, 64]. Due to the covalent nature of the hydrogen bond, a higher hydrogen concentration results in a less metallic bond and electrons localisation, in agreement with previous literature [4]. The extra hydrogen atom in PdH_0.3_ is marginal, since it does not significantly affect the bond character. For Pd and PdH (1:1), the primitive cell is used as the simulation box, whereas for PdH_0.25_, a supercell is used, effectively increasing the number of bands due to the system's symmetries.

**FIGURE 4:**
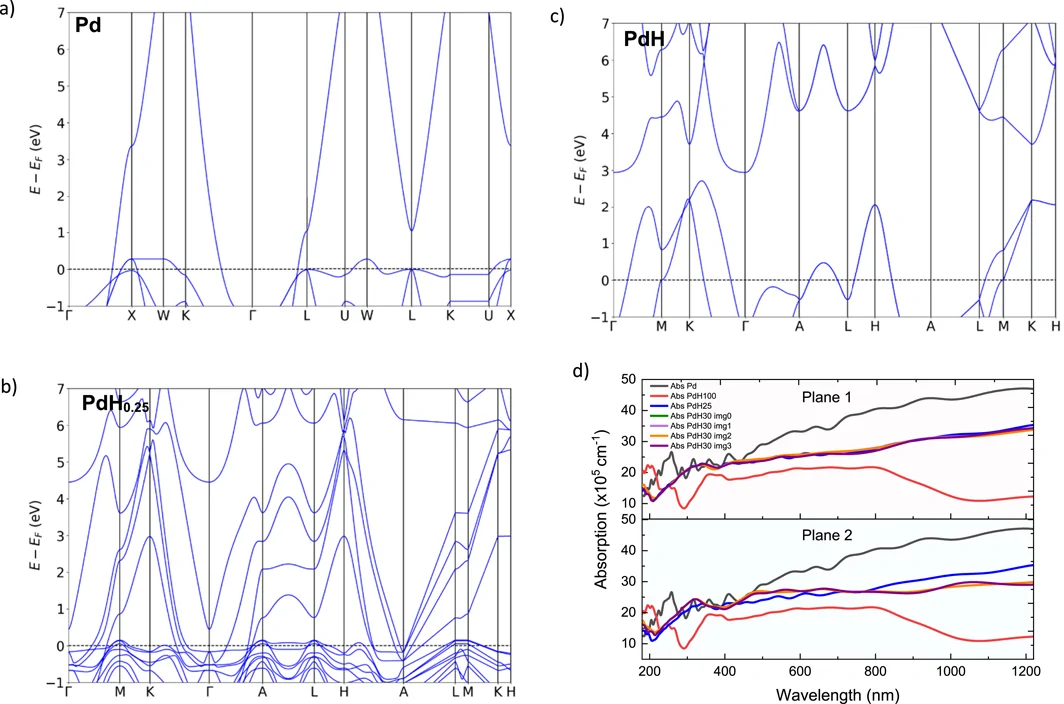
Band structures of (a) pristine palladium FCC (EF = −4.17 eV), (b) PdH_0.25_ (EF = −4.71 eV) and (c) PdH (1:1) (EF = −5.23 eV). The Fermi level is set as zero energy and is approximately at the same energy of the Pd d bands in the pristine case (a), above the Pd d bands for 25% H (b), and at much higher energy than the Pd d bands for the 100% H coverage (c) (d bands are not in range of the plot). (d) Absorption spectra for the planes 1 and 2 for Pd (black), PdH_0.25_ (blue), PdH (red) and PdH_0.30_ corresponding with different hydrogen positions as shown in the images in the Supporting Information S1: Figure S4a–c (green, pink, orange and purple). The optical absorption has decreased in the plane 2 for all PdH_0.30_ positions in the relevant wavelength range (450–700 nm).

The optical absorption spectra for Pd, PdH_0.25_ and PdH were calculated using band diagrams with the independent‐particle approximation (Figure 4d). This resulted in a decrease in absorption as a function of wavelength, which is consistent with previous studies [39, 65, 66]. However, the experimental and FDTD simulated spectra of Pd and PdH_x_ nanoparticle nanoparticulate thin films have a different dependence on the wavelength. This is due to the lack of nanostructures in the DFT calculations, which strongly affect the far‐field properties [8, 39, 67, 68] and increase diffusive reflection. This is confirmed by the FDTD simulations of Figure 1c, in which the increasing optical absorption from the literature is converted to a flatter spectrum with more features due to the nano‐structuring. Furthermore, the calculated optical absorption of PdH exhibits small peaks and valleys at approximately 1.2 and 4 eV, respectively. This can be explained by the high hydrogen concentration, which results in strongly localised bands from the 1s hydrogen orbital and 4d palladium orbital. Consequently, transitions between certain energy levels become more or less probable, giving rise to peaks in the absorption spectrum. Adding an “extra” hydrogen atom to PdH_0.25_ (to PdH_0.3_) does not significantly change the calculated absorption spectrum, since this increase in hydrogen concentration is insufficient to localise the bands.

To investigate the effect of a moving hydrogen atom on the optical absorption spectrum, a fifth hydrogen atom was added to the relaxed PdH_0.25_, resulting in PdH_0.3_. To determine the dynamics of that single atom, three possible trajectories were explored (Supporting Information S1: Figure S4a–c): the hydrogen plane between (1) the two top palladium layers, (2) the second and third palladium layer and (3) between these previous two planes. The calculated mean Fermi energies for the relaxed position (Supporting Information S1: Figure S4d) of each trajectory are, for plane 1: −4.68 eV, for plane 2: −4.65 eV and between planes: −4.68 eV.

Converting the energies corresponding to these hydrogen trajectories into absorption spectra for the planes 1 and 2 (Figure 4d) shows that adding an extra hydrogen atom (from PdH_0.25_ to PdH_0.3_) slightly increases optical absorption by ∼10% in the wavelength range from ∼450 to ∼700 nm, which is the one probed in our ultrafast spectroscopy measurements (Figure 2a–d). This absorption change of ∼10% provides a relative change in absorbed power for the 20 nm thick palladium film of ≈ 0.61%, using the Beer‐Lambert law [69, 70], which is in the order of magnitude of the persisting measured experimental nanosecond optical changes of ∼0.3% and therefore corroborates these findings. This result is obtained for plane 2 as well as for the in‐between plane (Supporting Information S1: Figure S5). Since adding extra hydrogen to plane 1 at any position within the plane does not change the optical absorption, it is confirmed that the position of the interstitial hydrogen, at a constant hydrogen concentration, affects its optical absorption. However, movement of hydrogen within the planes results in indistinguishable optical absorption curves, demonstrating that the experimentally observed optical changes are caused by inter‐plane hydrogen mobility, induced by the laser pump pulse. This can be explained by the relatively larger difference in distance between the mobile hydrogen atom and the hydrogen aggregate in the model. When the interstitial hydrogen atom is far away from other hydrogen atoms, it induces local optical properties corresponding to a low hydrogen concentration, resulting in minimal optical changes.

In general, the trend of the optical absorption of the palladium can be explained by the reduced likelihood of optical transitions between the conduction and higher valence bands, as obtained from the DOS. Although PdH is metallic, the absorption is lower because of the localisation of some of the orbitals of the palladium atoms, with additional peaks from the covalent character of the bond. Although the increased hydrogen concentration from PdH_0.25_ to PdH_0.30_ is not enough to result in a covalent character, it is enough to decrease the optical absorption slightly. Furthermore, considering the phase occurrence of *α* ≈ 52%, *β* ≈ 48%, it is likely that this absorption decrease is caused by the Pd‐H bonds in the unfilled β‐phase (which can therefore accommodate movement), which are more localised than the Pd‐Pd bonds, resulting in more localised DOS and rendering transitions between conduction and valence bands a less likely. For future research, a more detailed optical response could be obtained by scanning over the entire hydrogen concentration range, likely producing an optimal photon‐hydrogen interaction. Furthermore, the chromogenic materials such as yttrium hydride possess a much larger optical absorption change (contrast) depending on the hydrogen concentration [6, 71], making them highly interesting for follow up studies. The approach of this work, that is, the combination of ultrafast spectroscopy and chromogenic materials, can be extended to materials such as tungsten oxide [72] and vanadium oxide [73].

## 3 Conclusions

The intercalation of hydrogen inside a metal (palladium in this case), modifying its electric and optical properties, was investigated by ultrafast pump‐probe spectroscopy and DFT calculations, resulting in significant differences as compared to the palladium metal. The reduction of available free conduction band electrons, which are hybridised with the hydrogen atom s‐orbital, weakened the surface plasmon resonance, resulting in a shifting of the most intense optical transmission changes towards the red. The reduced surface plasmon oscillator strength also increased the damping of the oscillations due to an enhanced plasmon‐phonon coupling. Upon ultrafast photoexcitation, we observe a nearly instantaneous decrease of the sample transmission caused by the disruption of interstitial hydrogen atoms, which temporarily break their bonding with the palladium host. This results in an increased absorption due to a supply of free d‐band electrons released from the Pd‐H hybridisation, which persists over time scales much longer than picoseconds, in effect enabling the switching of light at various wavelengths by a single colour pump pulse. The here demonstrated fast but sufficiently lasting optical change, induced by turning the hydrogen “modulator”, forms a promising way to design responsive nanomaterials with photo‐switchable optical properties.

## 4 Experimental

The palladium (99.99% purity) nanoparticulate thin films of nominally 20 nm thickness were thermally evaporated on fused silica glass, which was cleaned with iso‐propanol and sonication. Hydrogenation was performed in a cylindrical aluminium cell of 20 cm diameter with Plexiglas windows which was evacuated to about 1 mbar after which ∼1 bar of Helium gas containing 15% hydrogen was let in. Previous investigations demonstrated that the maximum coverage of PdO at 300 K was 0.25 ML [74], allowing sufficient clean surface area for hydrogen to enter the nanoparticulate thin film [75].

The palladium nanoparticulate thin films were imaged by a Nanoscope III multimode scanning tunnelling microscope (STM) before and after hydrogenation (without (de)‐hydrogenation cycles). UV‐visible transmission spectra of the samples were recorded with a commercial double beam Agilent Cary‐100 spectrophotometer with a wavelength range from 190 to 900 nm, a wavelength resolution of 1 nm and a circular illumination spot with 5 mm diameter. The hydrogenated palladium nanoparticulate thin films were transported to the spectrometer in air in few minutes, which results in only a few percent optical absorption increase due to dehydrogenation occurring in the absence of a hydrogen atmosphere [8].

The ultrafast transient absorption (TA) spectroscopy experiments were performed by placing the samples inside a stainless steel chamber with optical windows, sealed by a valve, in which a pressure of around 1 mbar was reached using a diaphragm vacuum pump. The pristine samples were measured under vacuum while a hydrogen atmosphere (15% H_2_ and 85% He) was created to keep the hydrogen concentration inside the palladium nanoparticulate thin films constant. The chamber, specifically designed for ultrafast spectroscopy experiments, had a thin window to minimise dispersion‐induced stretching of the compressed pump pulse of approximately 20 fs time duration.

The ultrafast TA set‐up was based on a chirped pulse amplified Ti:Sapphire laser (Coherent Libra HE) that delivered a train of 100 fs pulses at a central wavelength of 800 nm (1.55 eV), with an average output power is 3 W with a pulse repetition rate of 2 kHz. This pulse train was divided into two beams from which the pump and probe pulses were generated, respectively. White light generated in a 1 mm thick sapphire crystal was used as probe pulse in the spectral range from 470 to 710 nm. The broadband pump pulse centred at 650 nm was obtained by compressing the output of a non‐collinear optical parametric amplifier (NOPA). The measurements were performed up to 80 ps pump‐probe delay controlled by a physical delay line. The pump was modulated using a mechanical chopper. The TA measurements were performed using a pump fluence of 120 µJ cm^−2^.

To understand and compare the static optical response of the palladium (hydride) nanoparticulate thin films, Finite Difference Time Domain (FDTD) simulations on commercial software (Lumerical‐Ansys) were performed using the literature values for the dielectric functions of Pd and PdH [8]. The evaporated granular palladium film was approximated by palladium spheres of 10 nm diameter on top of a thin glass layer.

To explain the observed ultrafast response of the PdH particulate film, calculations were performed using density functional theory (DFT) as implemented in the SIESTA code [59]. This method allows for efficient simulations of the electronic structure of materials using localised basis sets and norm‐conserving pseudopotentials with a low computational cost. Kohn‐Sham equations are solved using correlation and exchange interactions functionals as an approximation of the electron‐electron interaction. For the calculations, Local Density Approximation (LDA) with the Perdew‐Zunger (PZ) parametrisation were used. The electronic structure was described using a double‐zeta polarised (DZP) basis set, with two radial functions and an extra polarisation function. The Energy Shift from spatial confinement of the basis orbitals was set to 0.02 Ry and a SplitNorm contribution of the second zeta orbital of 0.15 was assumed. This basis set provides a good compromise between accuracy and computational efficiency.

The real‐space integration grid was defined by a MeshCutoff of 600 Ry, large enough to ensure convergence of total energy and electronic properties. The self‐consistent field (SCF) cycles were performed with 200 iterations as maximum, using six previous steps as Pulay mixing on the Hamiltonian and a mixing weight of 0.01. The density matrix convergence was set to 1.0·10^−4^ and the Hamiltonian to 1.0·10^−3^ eV. The Nudged Elastic Band (NEB) is a computational method used to find out the minimum energy path that connects two stable states.

The optical properties of the system were calculated with the independent‐particle approximation. The absorption spectra were obtained from the imaginary part of the dielectric function considering vertical transitions between occupied and unoccupied states of the calculated band structure. The SIESTA method uses three approximations to determine the optical properties. The imaginary dielectric function is adjusted by a Gaussian broadening; the polarisability of the system is approximated with the Kohn‐Sham orbitals and eigenvalues for excitation energies. The method does not include excitonic effects, electron‐hole interactions or phonon interactions.

## Author Contributions

**Floriana Morabito:** investigation, writing – review and editing, formal analysis, visualization, validation. **Paula H. Aguado:** investigation, writing – review and editing, formal analysis. **Luca Pedicone:** investigation, writing – review and editing, formal analysis, visualization, validation. **Alessandro Baserga:** investigation, writing – review and editing, formal analysis, validation. **Andrés R. Botello Méndez:** investigation, writing – review and editing, formal analysis, validation. **Stefano Dal Conte:** supervision, writing – review and editing, funding acquisition, resources, validation. **Christoph Gadermaier:** investigation, visualization, writing – review and editing, supervision, formal analysis, resources, validation. **Giulio Cerullo:** supervision, writing – review and editing, funding acquisition, resources, validation. **Zeila Zanolli:** investigation, funding acquisition, writing – review and editing, supervision, formal analysis, visualization, resources, validation. **Marcel Di Vece:** conceptualization, investigation, writing – original draft, methodology, validation, writing – review and editing, formal analysis, project administration, data curation, supervision, resources, visualization.

## Conflicts of Interest

The authors declare no conflicts of interest.

## Supporting information


Supporting Information S1

## Data Availability

The data that support the findings of this study are available from the corresponding author upon reasonable request.
