# Supplementary material for: Ultrafast Optical Hydrogen Modulator in a Palladium Hydride Nanoparticulate Thin Film
Source: Nanophotonics. 2026 Sep 6;15(17):e70288. doi: 10.1002/nap2.70288 (PMC13672179; doi:10.1002/nap2.70288)
Supplement: Supplementary file 1 [file NAP2-15-e70288-s001.pdf]

Supporting Information:

Ultrafast optical hydrogen modulator in a Palladium hydride thin film

Floriana Morabito[1], Paula H. Aguado[2], Luca Pedicone[3], Alessandro Baserga[1], Andrés R. Botello Méndez[2], Stefano Dal Conte[1], Christoph Gadermaier[1], Giulio Cerullo[1], Zeila Zanolli[2] and Marcel Di Vece[3]

[1] Dipartimento di Fisica, Politecnico di Milano, Piazza Leonardo da Vinci 32, I-20133 Milano, Italy

[2] Debye Institute for Nanomaterials Science, Utrecht University, Princetonplein 1, Utrecht 3584, The Netherlands;

[3] Interdisciplinary Centre for Nanostructured Materials and Interfaces (CIMaINa) and Physics Department "Aldo Pontremoli", Università degli Studi di Milano, Via Celoria 16, 20133, Milan, Italy

[*]Corresponding author: marcel.divece@unimi.it

Figure S1

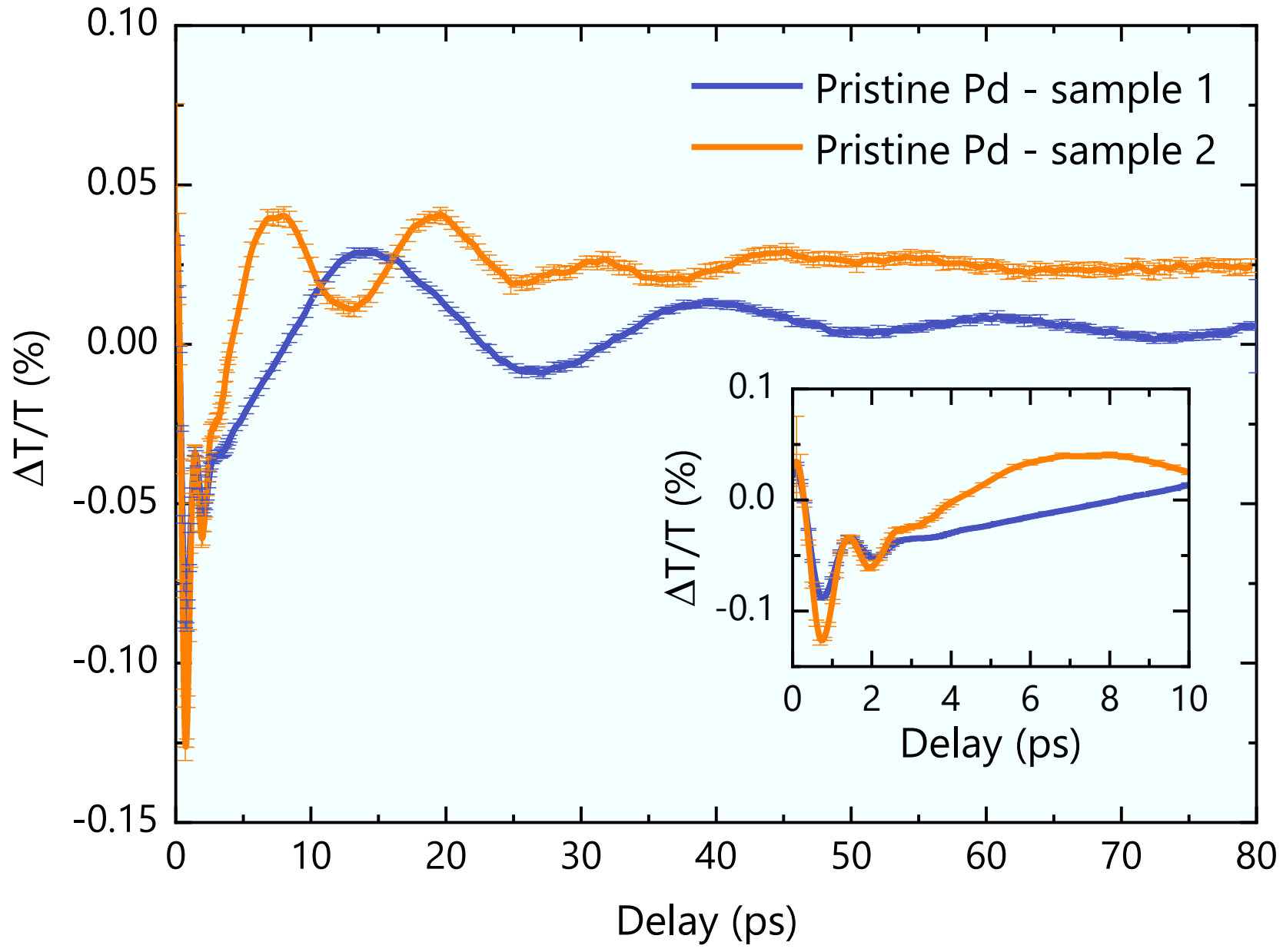

Effect of palladium thin film (~10nm) thickness on the phonon standing waves as result from the ps time scale electron-phonon dynamics. The oscillation period is longer for the thicker palladium film (~15nm) due to the red shift of standing wave. Pump: OPA 560 nm broadband, probed at 650±5 nm

Figure S2

a)

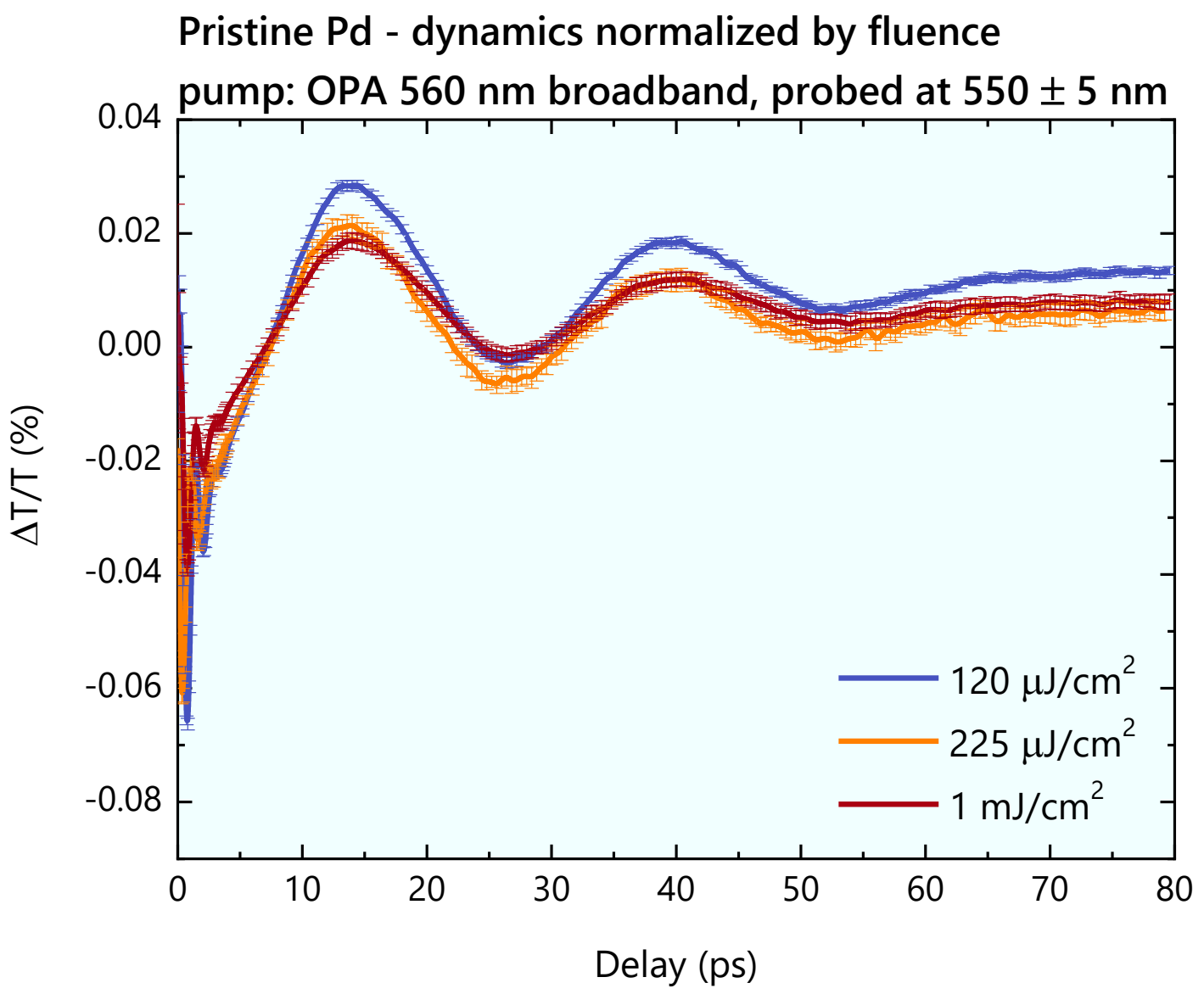

b)

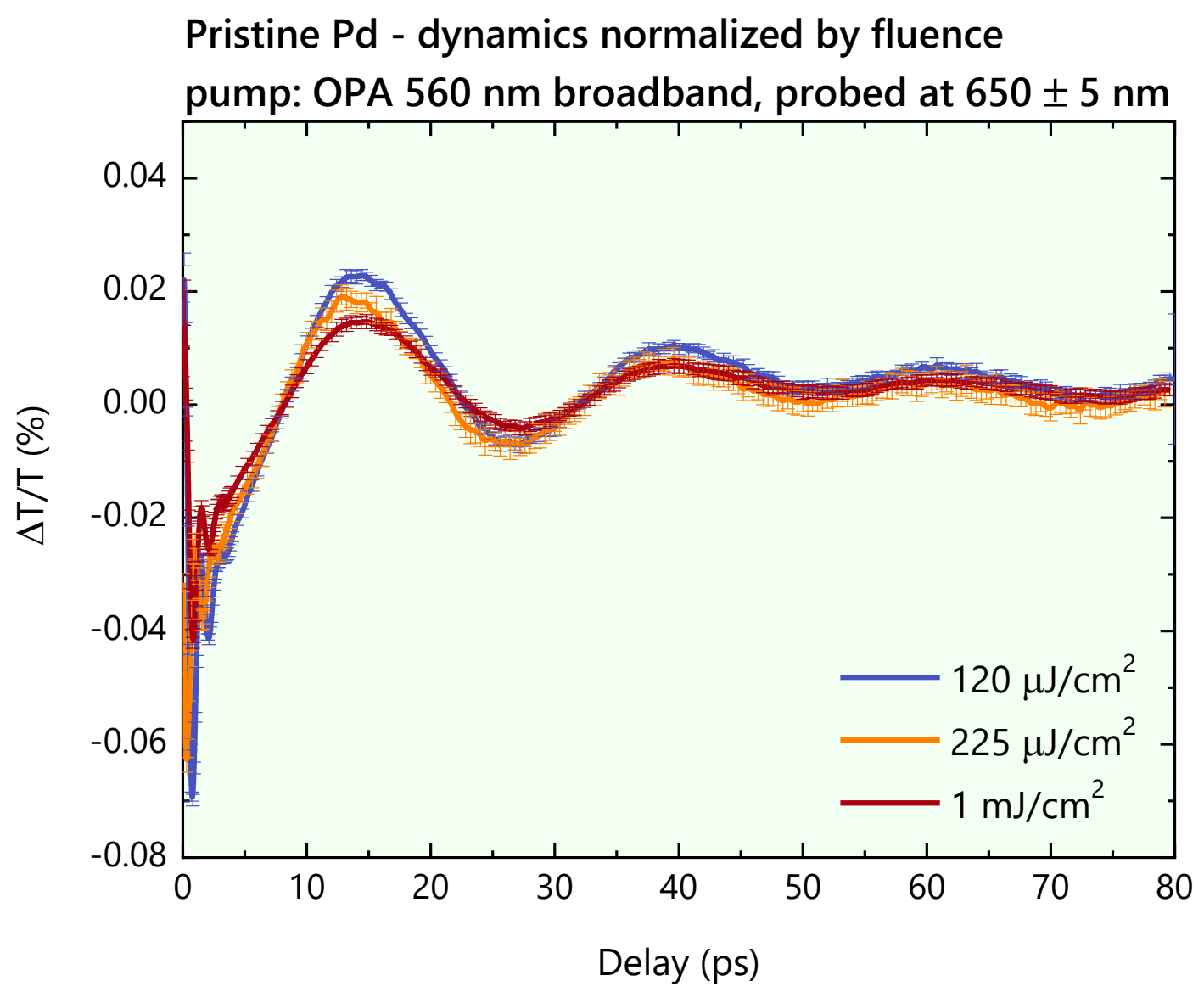

c)

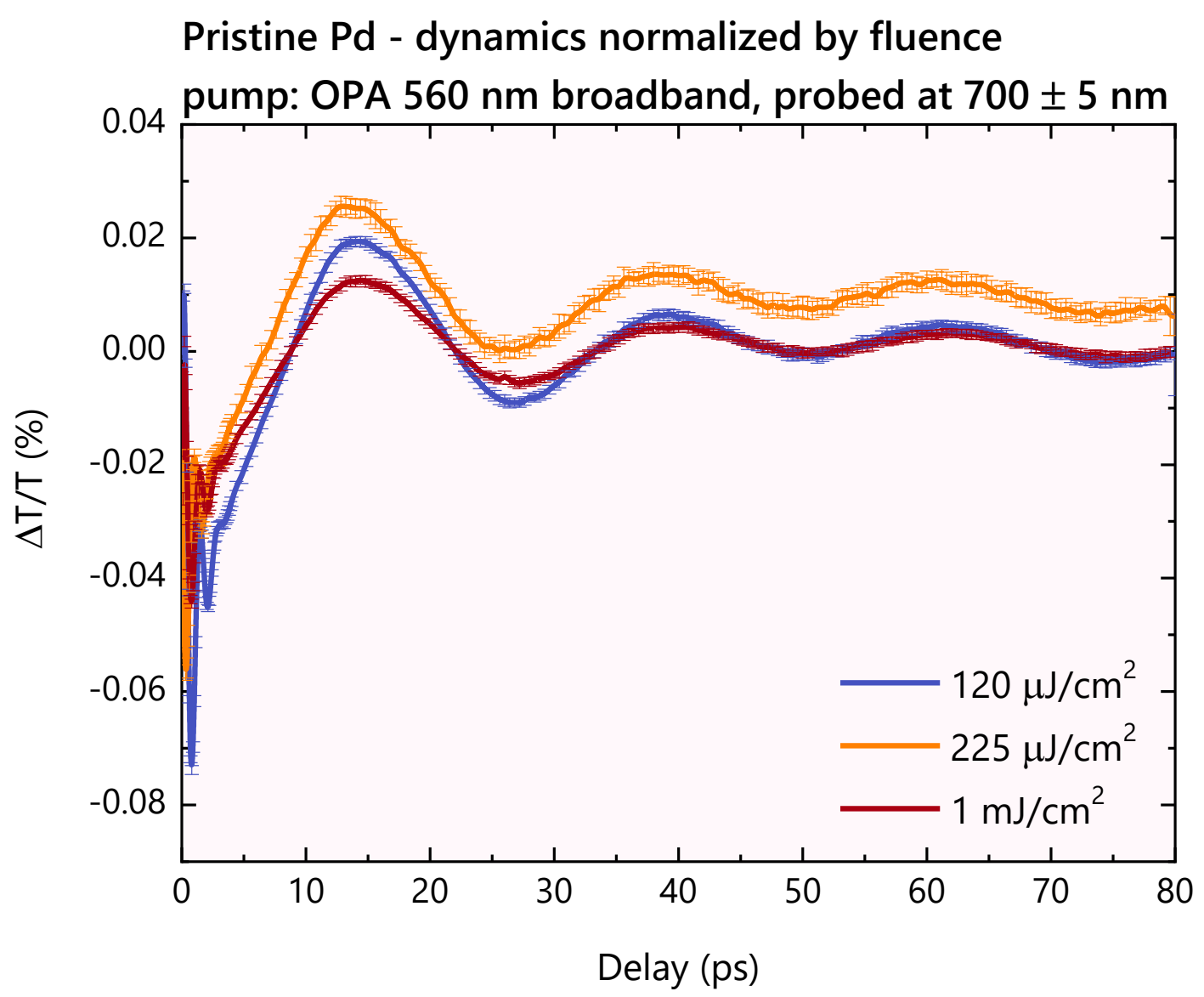

d)

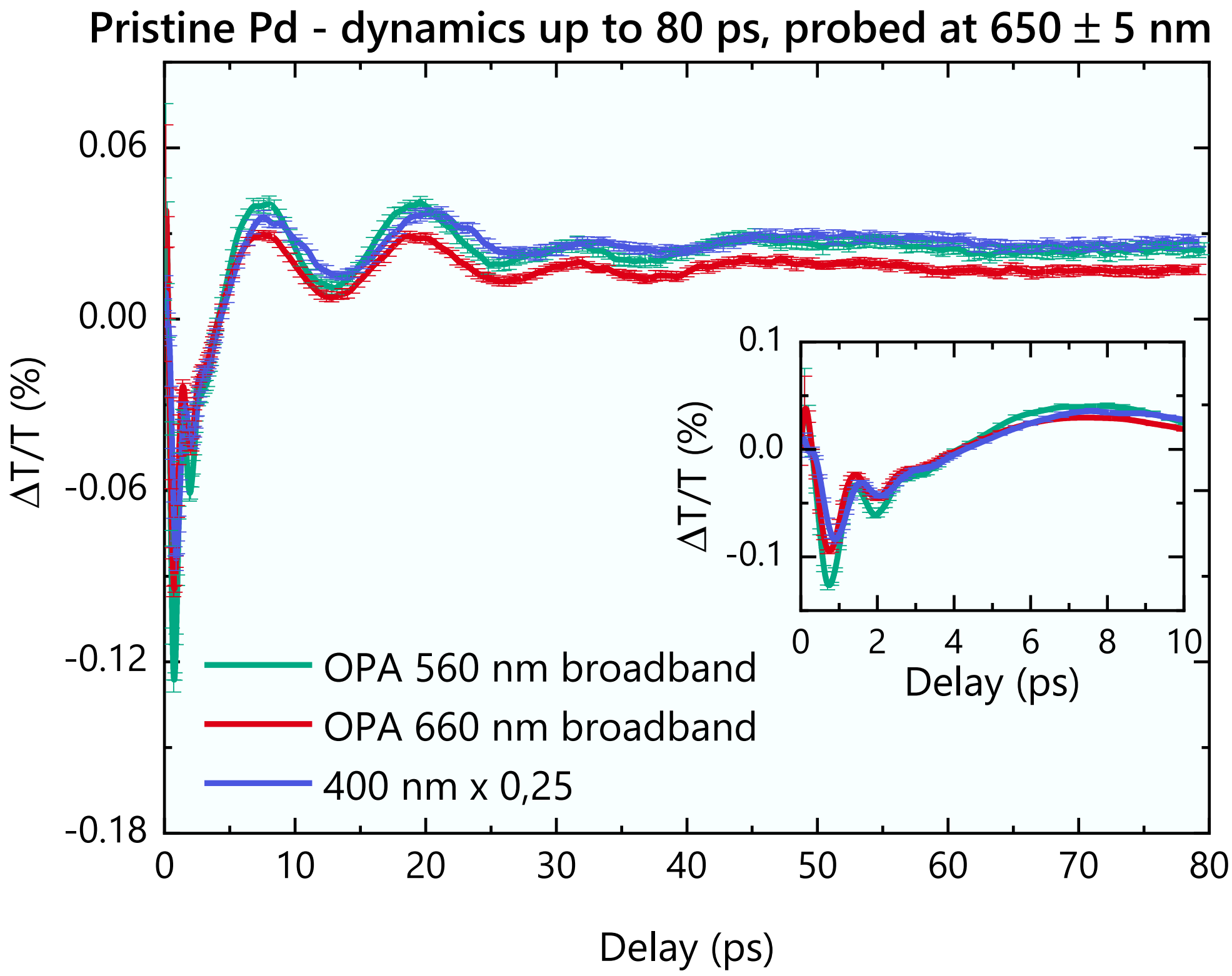

(a) : Comparison of the ΔT/T ultrafast dynamics up to 80 ps, normalised by fluence, for a pristine Pd thin film after 510 - 610 nm OPA broadband pump pulses with 120 μJ/cm², 225 μJ/cm² and 1000 μJ/cm² fluence values and probed at (a) 550 ± 5 nm, (b) 650 ± 5 nm, and (c) 700 ± 5 nm, demonstrating a fluence independent response (d) Pump-probe measurements of the palladium thin film under different wavelength ranges without significant differences, indicating a uniquely photon energy independence.

Parameters used in the $PdH_{0.3}$ calculations

Density of States (DOS) and Projected DOS (PDOS) calculations

To analyse the electronic structure, the projected density of states (PDOS) was calculated. For the PDOS calculation the k-points in the grid were increased to 100x100x100 to ensure an accurate integration over the Brillouin zone that leads in high resolution of the energies and a precise density of orbitals. The energy window calculated was (-1.0, 7.0) eV with a 0.02 eV resolution and a Gaussian broadening of 500 meV. The high value of the Gaussian broadening facilitates the interpretation of the DOS and allow us to identify the distribution of states associated with specific atomic orbitals in the system.

Optical properties calculations

The energy range for the optical spectra was set from -1.0 eV to 7.0 eV. Other parameters were converged as the Gaussian broadening in 0.1 eV and a k-grid of 50x50x25, for enough resolution of the absorbance plots. The polarization vector was defined along the z-axis, and to obtain an independent-orientation response a polycrystalline average was considered. All bands were used to ensure all available electronic transitions were included.

To compare the experiment and simulations, after the absorbance spectra were calculated, an average absorbance spectrum was calculated, using Boltzmann distribution.

$$\langle A \rangle = \sum_i \eta_i A_i \quad (4.3.1)$$

$$\eta_i = \frac{g_i e^{-\varepsilon_i / k_B T}}{Q} \quad (4.3.2)$$

$$\varepsilon_i = \Delta E_{tot/at} = E_{i/at} - E_{base/at} \quad (4.3.3)$$

Average absorbance, (4.3.2) Probability of position *i* and (4.3.3) Differential energy definition. $\mathbf{A_i}$ is the absorption spectrum of a certain image *i*, $\boldsymbol{\eta_i}$ is the probability of a certain image i, $\mathbf{g_i}$ is the multiplicity of a certain image i, $\boldsymbol{\varepsilon_i}$ is the differential energy between the image *i* and a base line, **Q** is the partition function of the system, $\mathbf{K_b}$ is Boltzmann constant and **T** is the temperature.

The average spectra are calculated using as the base-line the less energetic configuration of the $PdH_{0.3}$ system.

Figure S3

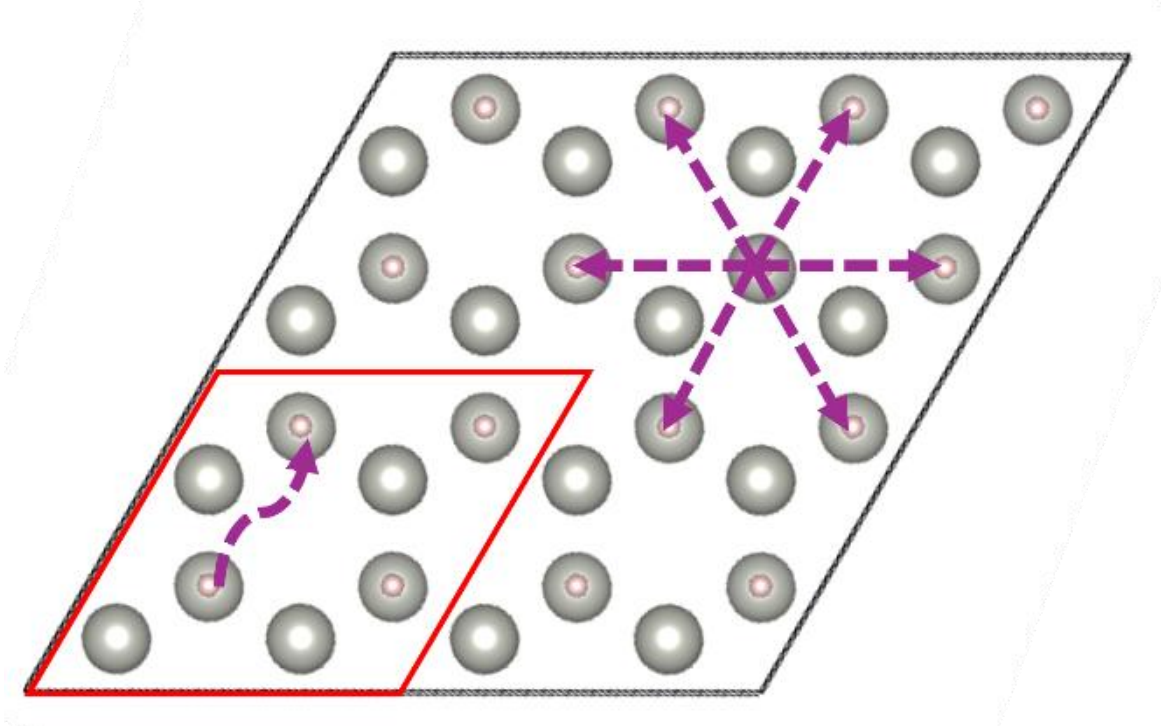

$PdH_{0.3}$ Scheme to determine the multiplicity of each image ion NEB trajectories. Palladium atoms (silver) and Hydrogen atoms (pink).

The multiplicity of the image *i* is defined as the number of equivalent images the system will have if the final point of the NEB calculation changes to another relaxed position. The multiplicity of the images is determined in Fig. 3: for Plane 1 trajectory g_i is six. For Plane 2 and Between Planes trajectories g_i is twelve since there are two equivalent “plane 2” considering PBC.

The temperature used to calculate the spectrum was 25 ºC (297.15 K) since the experimental spectra were measured at room temperature.

$PdH_{0.3}$ relaxed NEB trajectories

Three possibles paths were studied as shown below:

Figure S4

a) Plane 1

b) Plane 2

c) Between planes

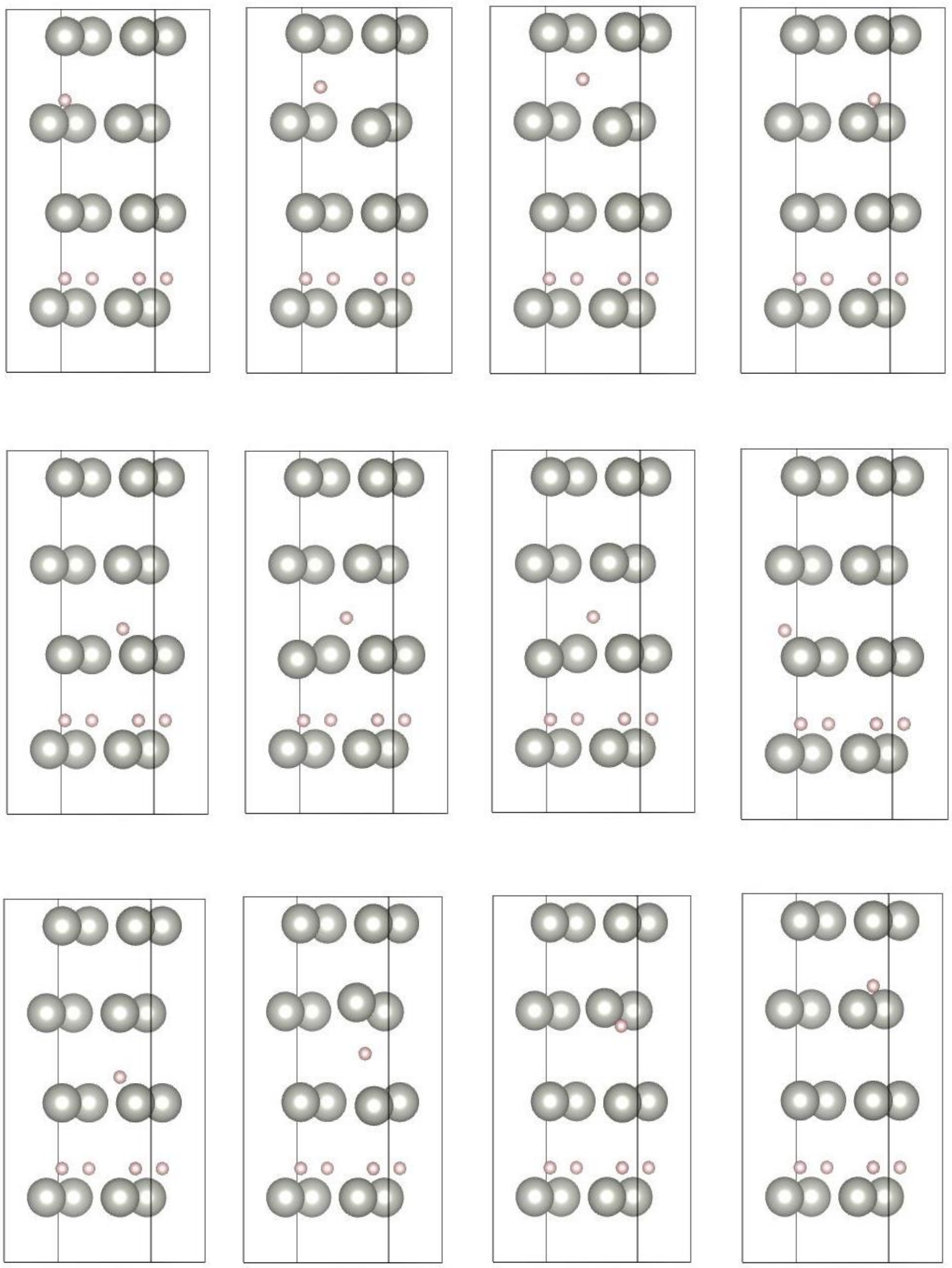

Relaxed images within (a) plane 1, (b) plane 2 and (c) Between Planes, from left to right: Image 0, Image 1, Image 2, and Image 3. Palladium atoms (silver) and Hydrogen atoms (pink). (d) Energy profiles of hydrogen between plane trajectories

d)

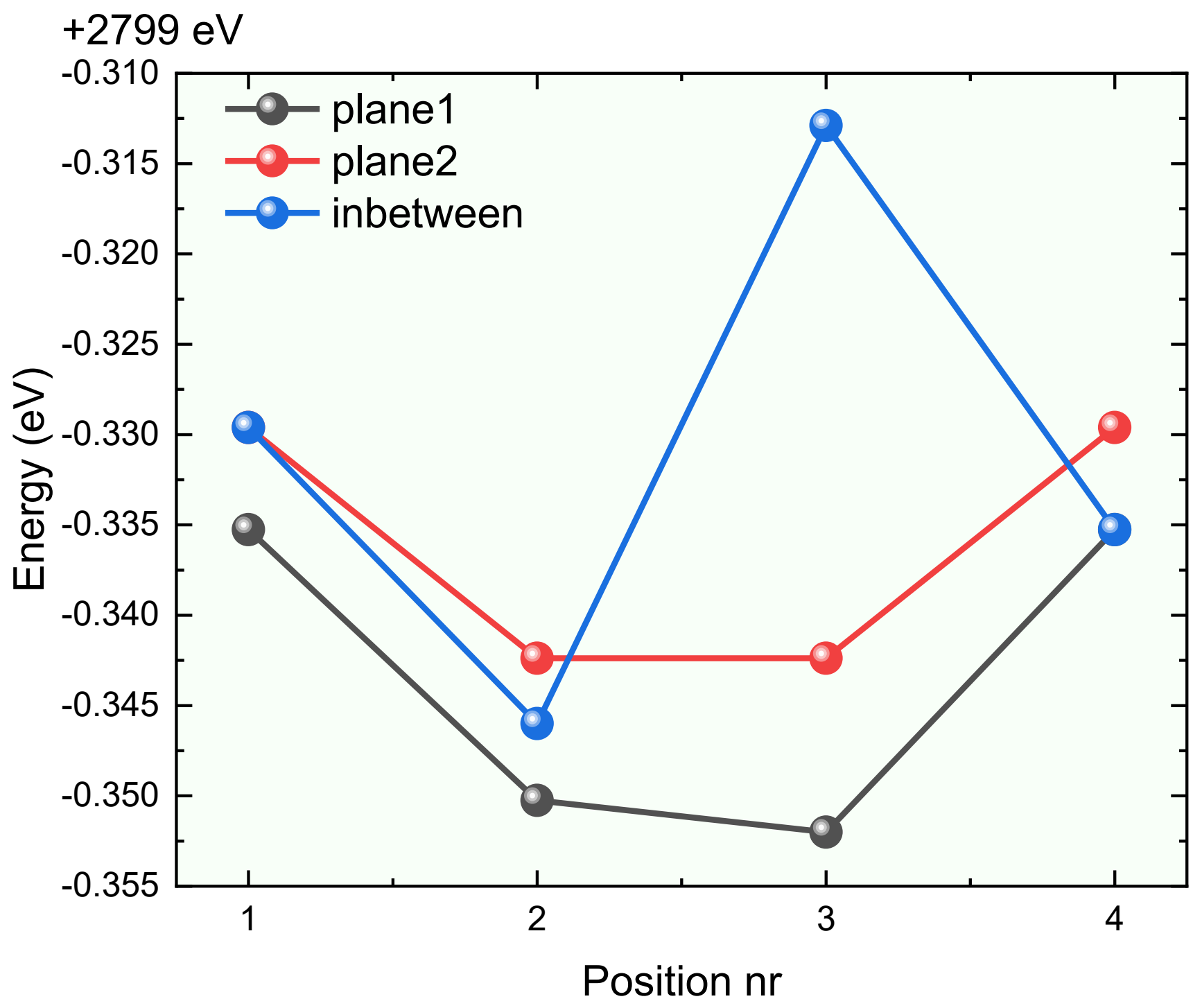

When the interstitial hydrogen atom is located around the middle point between the palladium planes, the surface potential is smoother and the hydrogen atom can move easily, namely the energy barrier is lower, viewing from the potential energy surface along the plane perpendicular to the c axe for each of the images. For Plane 2, the relaxed hydrogen position converges in only three images, since Image 1 and Image 2 are the same; and have the same energy. As in Plane 1, the inner image is less energetic than the extrema, due to the smoothness of the potential curve. The in “Between Planes” trajectory, is more stable although Image 2 has higher energy since the hydrogen atom is closer to the palladium atoms layer with different potential energy surface: Image 1 is in a smooth area of the surface, while Image 2 is located in a in a high potential energy area.

DOS and PDOS

According to the calculated density of states graphs optical properties can be predicted. On the one hand, for all the systems studied, the density of states is high, just below the Fermi level, while the number of states begin to decrease for energies above the Fermi level. For an optical absorption spectrum, a higher absorption at lower energies is expected, near infrared (NIR) and visible range (> 780 nm) and lower absorption at higher energies. On the other hand, the hydrogen PDOS has a higher number of bands at higher energies, which leads to higher energy electronic transitions. Although transitions at high energies, Ultraviolet (UV) range (< 780 nm), are expected, these transitions will be considerably less frequent, than lower energy transitions, since the hydrogen contribution to the DOS is relatively low.

Absorption spectra of $PdH_{0.3}$ in NEB calculations

Converting the energies corresponding to the hydrogen trajectories into absorption spectra for the planes 1, 2 and in-between (Figure 5) shows that adding an extra hydrogen atom (from $PdH_{0.25}$ to $PdH_{0.3}$) slightly increases optical absorption by ~10% in the wavelength range from ~450nm to ~700nm for the plane 2 and in-between planes, which is the one probed in our ultrafast spectroscopy measurements.

Figure S5

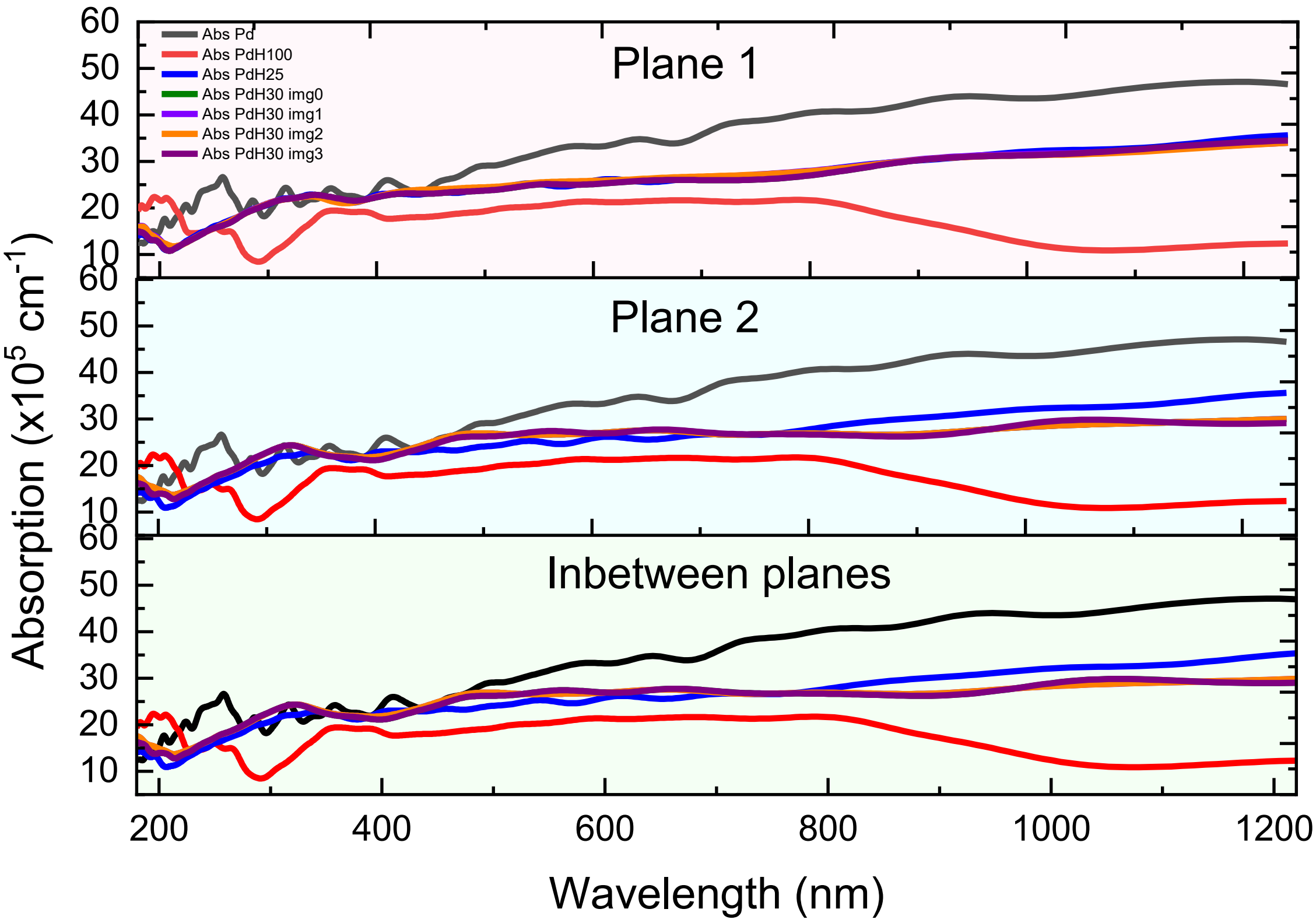

Absorption spectra of PdH_x for Planes 1 and 2 and in between for different trajectories as obtained from the band structures (see article).

Figure S6

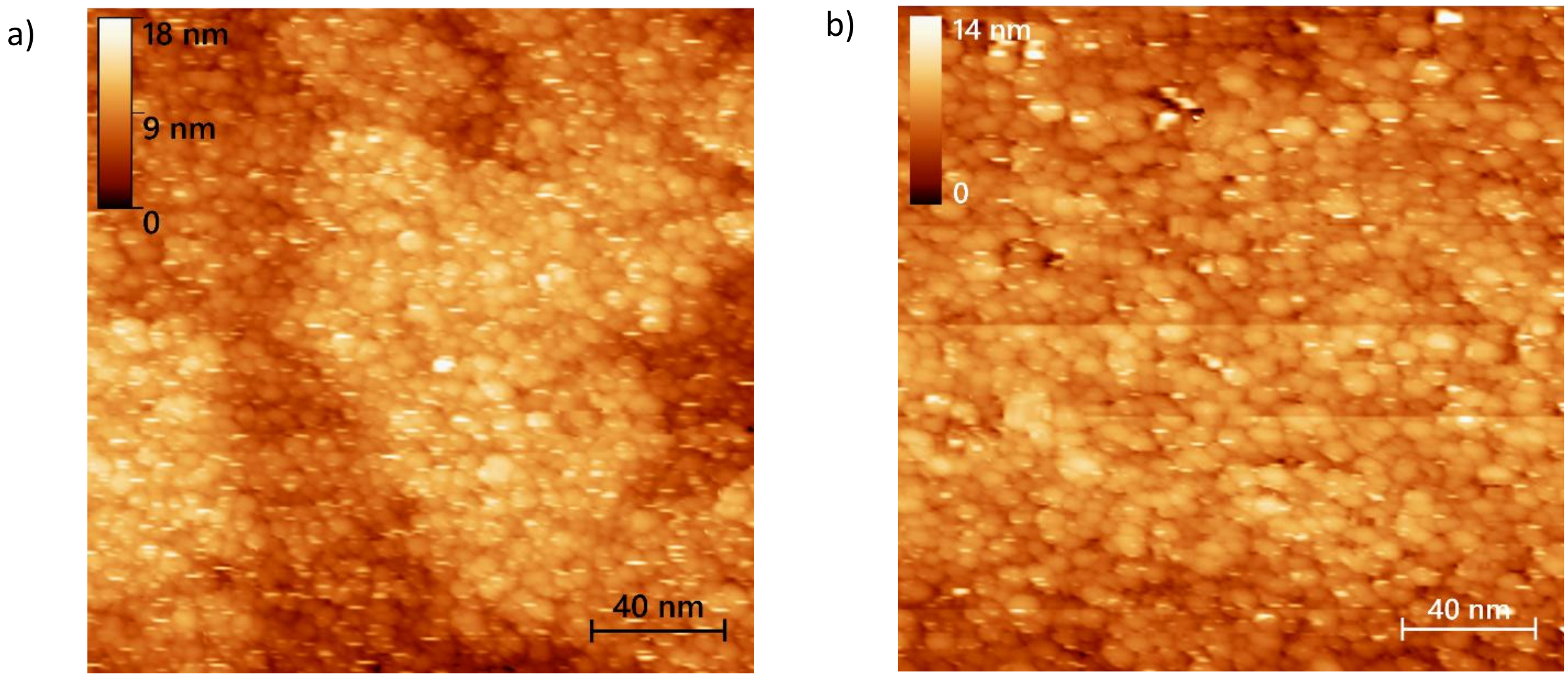

An image STM micrograph of the (a) pristine palladium thin film with spherical nanostructures clearly visible as they constitute the Pd film. (b) An image of the Pd thin film post hydrogen absorption. (c) The hydrogenated Pd NPs are centred on a slightly higher value (6.6 ± 0.8 nm) than the one in the pristine state (5.7 ± 0.7 nm).
